# Discovery of MLL1 binding units, their localization to CpG Islands, and their potential function in mitotic chromatin

**DOI:** 10.1186/1471-2164-14-927

**Published:** 2013-12-28

**Authors:** Minou Bina, Phillip Wyss, Elise Novorolsky, Noorfatin Zulkelfi, Jing Xue, Randi Price, Matthew Fay, Zach Gutmann, Brian Fogler, Daidong Wang

**Affiliations:** 1 Department of Chemistry, Purdue University, West Lafayette, IN 47907, USA

**Keywords:** *Cis*-elements, Chromatin structure, Codes in DNA, CGG repeats, CpG islands, FMR1, HOXA, HOXB, HOXC, HOXD, MLL, MLL1, Gene bookmarking, Gene regulation, Human genome, Mammalian genomes, Regulatory codes, Trithorax response elements, TREs, Mitosis, Cell division

## Abstract

**Background:**

Mixed Lineage Leukemia 1 (MLL1) is a mammalian ortholog of the *Drosophila* Trithorax. In *Drosophila*, Trithorax complexes transmit the memory of active genes to daughter cells through interactions with Trithorax Response Elements (TREs). However, despite their functional importance, nothing is known about sequence features that may act as TREs in mammalian genomic DNA.

**Results:**

By analyzing results of reported DNA binding assays, we identified several CpG rich motifs as potential MLL1 binding units (defined as morphemes). We find that these morphemes are dispersed within a relatively large collection of human promoter sequences and appear densely packed near transcription start sites of protein-coding genes. Genome wide analyses localized frequent morpheme occurrences to CpG islands. In the human *HOX* loci, the morphemes are spread across CpG islands and in some cases tail into the surrounding shores and shelves of the islands. By analyzing results of chromatin immunoprecipitation assays, we found a connection between morpheme occurrences, CpG islands, and chromatin segments reported to be associated with MLL1. Furthermore, we found a correspondence of reported MLL1-driven “bookmarked” regions in chromatin to frequent occurrences of MLL1 morphemes in CpG islands.

**Conclusion:**

Our results implicate the MLL1 morphemes in sequence-features that define the mammalian TREs and provide a novel function for CpG islands. Apparently, our findings offer the first evidence for existence of potential TREs in mammalian genomic DNA and the first evidence for a connection between CpG islands and gene-bookmarking by MLL1 to transmit the memory of highly active genes during mitosis. Our results further suggest a role for overlapping morphemes in producing closely packed and multiple MLL1 binding events in genomic DNA so that MLL1 molecules could interact and reside simultaneously on extended potential transcriptional maintenance elements in human chromosomes to transmit the memory of highly active genes during mitosis.

## Background

The DNA in human chromosomes is relatively long [1]. In addition to protein-coding genes, the genome includes numerous sequence features including gene deserts [2], a multitude of long noncoding RNAs with little or no protein-coding capacity [3], and many islands of CpG-rich sequences [4]. CpG Islands (GIs) include G-tracts and numerous nonmethylated CpGs [4]. CpG-richness is a remarkable feature since, generally, bulk genomic DNA is depleted of CpG, owing to selective deamination of 5-meC [5,6]. CGIs vary in size and CpG content [6-8]. In close proximity (~2 kb) to CGIs, there are regions (known as shores) that contain a lower CpG density than the values computationally selected to define the position of CpG islands [9,10]. Sequences (~2 kb) that flank the shores are referred to as shelves [11]. Sequences beyond the shelves are described as open sea [11]. Both shores and shelves appear to contribute to developmental and regulatory processes that control CpG methylation patterns in chromosomes leading to gene repression [12].

Gene repression and activation are regulated by proteins that interact with DNA, by enzymes that modify the core histones in nucleosomes and by proteins that bind modified residues in histones [13]. Core-histone modifications include methylation (me), acetylation (ac), phosphorylation (P), and ubiquitination (ub) [14]. A conserved domain (SET) catalyzes methylation of H3K4 (lysine 4 in histone H3) producing H3K4me3 [15]. Trimethylated H3K4 is associated with active or transcriptionally poised chromatin states [16]. In mammalian cells, H3K4 trimethylation involves several enzymes that include SETD1A, SET1DB, and members of MLL family. MLL family members are comprised of MLL1, MLL2, MLL3, and MLL4 [15,17]. In the literature, the human MLL1 is also referred to as MLL, ALL-1, and MLLT1; its official symbol is KMT2A. In our studies, we refer to human KMT2B as MLL2, to KMT2C as MLL3, and to KMT2D as MLL4.

Earlier studies discovered the *MLL1* gene through its involvement in chromosome translocations that cause acute leukemia [18,19]. Translocations often produce abnormal proteins consisting of the amino-terminus of MLL1 fused in frame to the carboxyl terminus of another protein [20]. The normal form of MLL1 is relatively large and contains several domains: a plant homeodomain, a bromo domain, a transactivation domain, a SET domain, and a cysteine-rich CXXC domain [21]. The CXXC domain is known as MT since it shows sequence similarity to DNA methyltransferases [22,23]. A similar domain exists in MLL2 and CXXC1 (also known as CGBP and CFP1). Even though the MT domain in MLL1 and CXXC1 binds non-methylated CpG containing sequences [24-26], swapping experiments have shown that CXXC domains have specific and nonredundant activities that impact downstream regulatory functions [27]. Colony forming ability and leukemogenicity of a fusion protein (MLL-AF9) was abrogated when the MLL-derived segment was replaced with the DNA binding domain of CXXC1 [27]. Furthermore, even though MLL1 and MLL2 displayed almost indistinguishable DNA-binding properties, their corresponding MT-domains guided the proteins to largely non-overlapping gene repertoires [25].

Evidence supports central roles for native forms of MLL1 in mechanisms that preserve “the memory” of highly active genes during cell division and at specific stages in embryonic development [28-31]. In *Drosophila*, two groups of proteins support heritable memory systems that maintain the transcriptional state of target genes [32,33]. Trithorax Group (TrxG) binds TrxG Response Elements (TREs) to maintain active states [32]. Polycomb Group (PcG) perpetuates repressed states through PcG Response Elements (PREs) [32,33]. In *Drosophila*, related DNA sequence elements are thought to contribute to the recruitment of both TrxG and PcG complexes to chromatin [32]. Mammalian PcG proteins consist of two groups: Polycomb Repressive Complexes 1 and 2 (PRC1 and PRC2), see [34] and references therein. PRC1 catalyzes mono-ubiquitylation of histone H2A; PRC2 methylates lysine 27 in histone H3 producing H3K27me2/me3 [16,35]. The PRC2 complex includes EZH2, EED, and SUZ12 [36]. EZH2 is the enzymatic component of the PRC2 complex and produces the repressive H3K27me3 marks in nucleosomes [16,35]. Interestingly, emerging data indicate that the PRC2 complex is recruited to chromatin by CpG islands [34].

Syndromic manifestations support the opposing functions that MLL1 and EZH2 play in embryonic development. Mutations in the *EZH2* gene cause autosomal dominant Weaver syndrome characterized by generalized overgrowth, advanced bone age, marked macrocephaly, hypertelorism, and characteristic facial features [37,38]. *De novo* mutations in the *MLL1* gene cause Wiedemann-Steiner syndrome [39-41]. Symptoms vary and may include delayed growth and development, asymmetry of the face, hypotonia, and intellectual disability [39-41]. Mutations often produce frame-shifts removing downstream domains. Studies of *Mll1* knockout mice support a central role for MLL1 in regulating developmental pathways [28-30]. *Mll1* heterozygous (+/-) mice displayed retarded growth, haematopoietic abnormalities, and bidirectional homeotic transformations of the axial skeleton as well as sternal malformations [28]. *Mll1* deficiency (-/-) was embryonic lethal [28]. In mice, *Mll1* was required for maintaining gene expression early in embryogenesis [42], necessary for correct development of multiple tissues, and essential for successful skeletal and neural, and craniofacial development [28,42].

**Figure 1:**
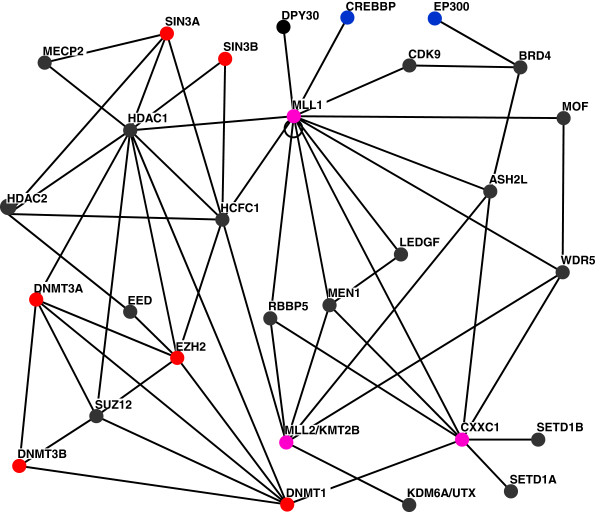
**A subset of protein networks that involve MLL1.** Proteins are depicted as nodes, their interactions as edges [44]: pink, proteins that bind unmethylated CpGs; red, proteins found in repressive complexes. The interactions were obtained from BioGRID [45,46]. The figure does not display all reported interactions. It focuses on underlying connectivity among proteins that associate with MLL1 to form large and highly dynamic multiprotein complexes.

Protein networks that include MLL1 drive coordinated patterns of gene expression (Figure 1). These networks are organized as hubs that receive and transmit information to activate, upregulate, downregulate, or repress the expression of a given gene [13]. Components in molecular circuitries include multiprotein complexes that are relatively large and highly dynamic [13]. Depending on environmental milieu, MLL1 associates directly or indirectly with numerous regulatory proteins including MEN1, RBBP5, WDR5, ASH2L, HCF1, LEDGF, and CXXC1 (Figure 1). In protein networks, MLL1, HCF1, and CXXC1 also communicate with large and dynamic protein complexes that repress transcription (Figure 1). CXXC1 binds non-methylated CpG [26,43] and interacts with H3K4 methyltransferases known as SET1A/ SETD1A and SET1B/ SETD1B (Figure 1). These enzymes play a more widespread role in H3K4 trimethylation than do MLL1 complexes in mammalian cells [17]. These and related findings indicate that in addition to H3K4 methylation, MLL1 performs histone methyltransferase-independent functions [31].

As the main component in trithorax-based regulation networks, MLL1 plays a central role in preserving transcriptional memory during mitosis [31]. Analyses of synchronized human cells identified a globally rearranged pattern of MLL1 occupancy during mitosis in a manner favoring genes that were highly transcribed during the interphase stage of cell-cycle [31]. However, how MLL1 bookmarks genes to maintain transcriptional memory has not been addressed. The finding that gene-bookmarking by MLL1 is largely independent of the methylation status of H3K4 on mitotic chromosomes [31] provokes the question of whether interactions of MLL1 with genomic DNA may play a role in bookmarking events that preserve the memory of highly transcribed genes at the onset of mitosis. To explore this question, we have analyzed data concerning interactions of MLL1 with DNA and chromatin. We show that DNA sequences that bind the MLL1 MT-domain can be described as minimal units or morphemes: the smallest ‘words’ in DNA that selectively bind the MT-domain in MLL1. We find that the MLL1 morphemes occur in chromatin segments that are bookmarked by MLL1 during mitosis. Furthermore, we show that frequent morpheme occurrences map to genomic sequences that correspond to CGIs. Collectively, our results suggest that CGIs include TREs that bind MLL1 to maintain the memory of highly active genes at the onset of mitosis.

## Results and discussion

### Localization of CpG-rich motifs in promoters of human protein-coding genes

Protein coding genes are transcribed by RNA polymerase II (POLII). Earlier studies deduced that MLL1 exclusively regulated the expression of homeotic genes and proper segmental identity in mammals [28,42]. However, emerging data indicate that MLL1 associates with a substantial fraction of human POLII promoters, supporting a global role for MLL1 in regulation of transcription [31,47].

**Figure 2:**
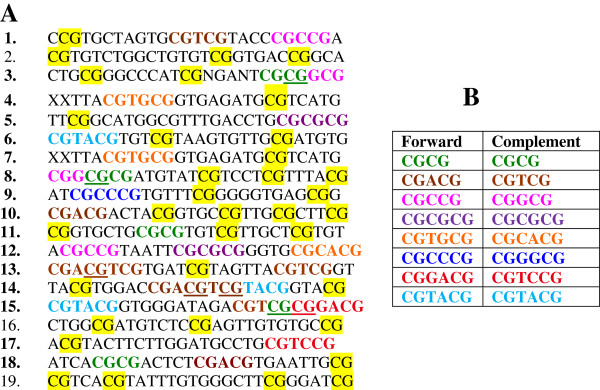
**Analysis of cloned inserts obtained from SELEX assays. (A)** The inserts were isolated and sequenced by Birke et al. [24]. We numbered the inserts as shown on the left. Bold numbers highlight inserts that include one or more MLL1 morpheme(s). Underlined CGs denote the position of morpheme overlaps. Yellow boxes highlight CpGs that did not correspond to discernable motifs. **(B)** Color-coding scheme for distinguishing various MLL1 morphemes.

To uncover sequence motifs that may selectively interact with MLL1, we analyzed sequences of 19 cloned inserts that the MT-domain in MLL1 selected in DNA binding assays [24]. In 16 inserts, we identified motifs consisting of CGCG with 0–2 nucleotides between the two CpGs. The remaining 3 inserts contained CpG but lacked discernable motifs (Figure 2A). To explore the relevance of the identified motifs to gene regulation, we examined a relatively large collection of human POLII promoters. We focused on the region upstream of transcription start sites (-500 to -1) since this DNA segment contributed to formation of protein complexes that regulated initiation of mRNA synthesis [13]. In promoter selection, we imposed filtering criteria to eliminate redundancy. The final promoter set included nearly 16,000 sequences. We analyzed this set for occurrences of CGCG, CGNCG, and CGNNCG. Additional file 1: Figure S1 shows that these motifs are spread across the DNA segment that precedes the transcription start sites (TSSs). Motif frequencies steadily increase in sequences approaching proximal promoters and TSSs in genomic DNA (Additional file 1: Figure S1). Certain motifs appear more prevalent than others, displaying the following trend: CGNNCG > CGNCG > CGCG.

### Lexical units recognized by the MLL1 MT-domain and their localization to POLII promoters

Encouraged by results of preliminary promoter analyses, we asked whether the cloned inserts obtained from SELEX assays included sequence-elements that may correspond to MLL1 recognition sites. To approach this question, we separated motifs consisting of CGNCG and CGNNCG according to nucleotides that appeared at N position. We uncovered several motifs, which we refer to equivalently as MLL1 binding sites, binding units, or morphemes (Figure 2B). Examples include CGCG, CGTCG or its complement (CGACG), CGGCG or its complement (CGCCG), and CGTACG, a palindromic sequence (Figure 2B). Thus, the MLL1 morphemes derived from CGNCG include all possible bases at the N position: A, G, C, or T. Among the combinatorial permutations of NN (in CGNNCG), we did not find CGGCCG, CGAACG, CGATCG, CGAGCG, and CGACCG. We refer to these sequences as non-motifs.

Results of promoter analyses prompted examination of a sequence pattern that appeared frequently at the 5′ boundary of human POLII genes [48]. This pattern consists of BVSCGSSSCB: where B corresponds to C, G, or T; V to A, C, or G; S to C or G. We find that this pattern describes three of the MLL1 morphemes (CGCGCG, CGCCCG, CGCCG), supporting a possible role for such morphemes in regulation of transcription. Additionally, earlier studies analyzed human POLII promoters for frequently occurring 8-mers and 9-mers [49-52]. When ranked according to statistical criteria, including occurrences in total human genomic DNA, we find that a relatively large proportion of promoter 9-mers are composed of CpG-rich sequences [49,50] that include MLL1 morphemes.

**Figure 3:**
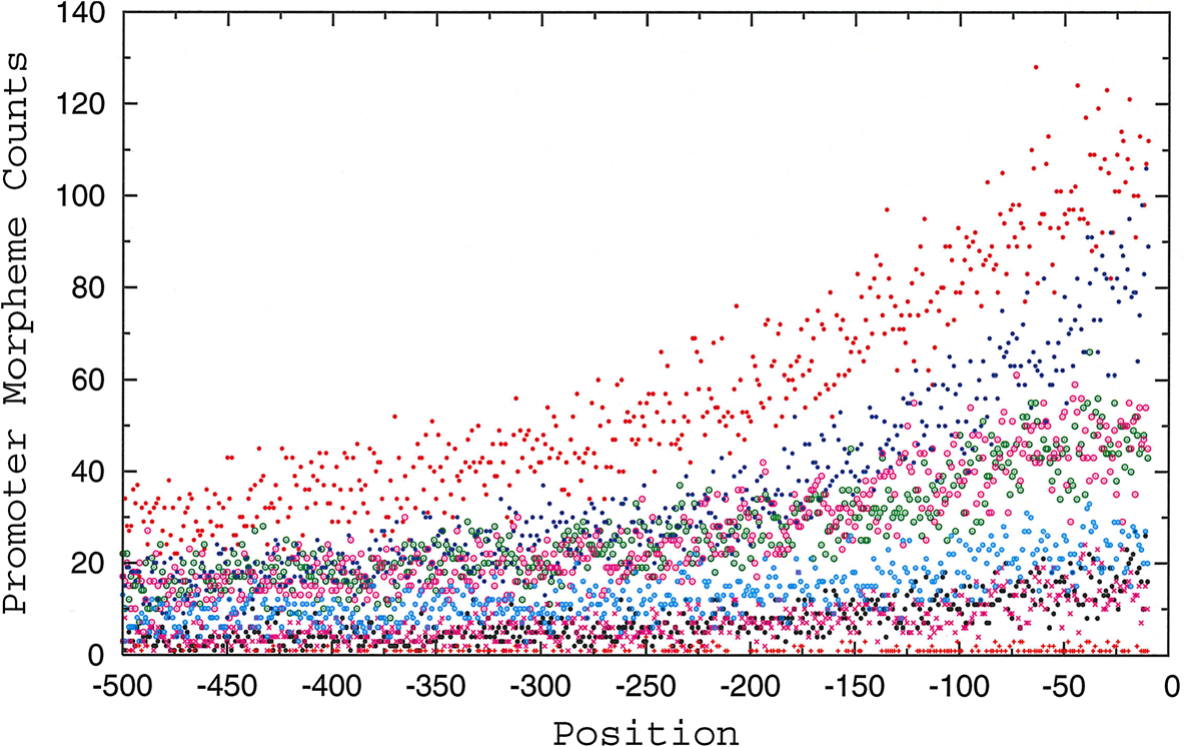
MLL1 morpheme distribution and occurrences in promoters of human protein-coding genes: CGCG, red full circles; CGGCG, blue full circles; CGCCCG, magenta empty circles; CGACG, green empty circles; CGGACG, light blue empty circles; CGCGCG, black circles; CGTGCG, x magenta; CGTACG, + red.

Therefore, we reanalyzed the POLII promoter set for morpheme occurrences (Figure 3, Additional file 2: Table S1). We find that as observed for CGCG, CGNCG, and CGNNCG (Additional file 1: Figure S1), the MLL1 morphemes are spread across POLII promoters and their density increases in sequences approaching the TSSs (Figure 3). For morpheme frequencies, we observe the following trend: CGCG > CGGCG > CGACG > CGCCCG > CGGACG > CGCGCG > CGTGCG > CGTACG (Additional file 2: Table S1). *In toto*, results of complementary analyses support a role for MLL1 morphemes in promoter-associated functions.

### Morpheme occurrences in functional DNA sequences

**Figure 4:**
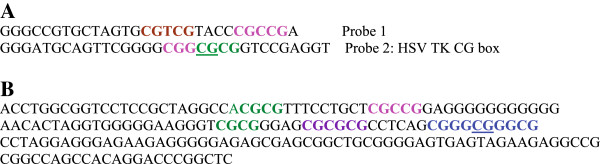
**Morphemes in DNA fragments that interact with MLL1 in DNA binding and functional assays. (A)** Sequences analyzed in DNA binding or transient expression assays. Probe 1 corresponded to insert 1, shown in Figure 2A; probe 2 was derived from the HSV TK promoter [53]. Colored sequences highlight the position of MLL1 morphemes in probe 1 and probe 2 **(B)** MLL1 morphemes in a DNA segment from the mouse *Hoxa9* gene. This segment includes the promoter of a *Hoxa9* transcript [54]. Colored sequences highlight CpG-rich clusters that *in vivo* MLL1 protected from methylation [54]. Color-coding follows the scheme in Figure 2B.

Since the MLL1 morphemes were identified from the results of SELEX assays, we asked whether the morphemes have any relevance to sequences that bind MLL1 in a cellular context. In literature surveys, we found studies that dealt with interactions of MLL1 with both synthetic and naturally occurring DNA sequences [53-55]. One study examined a naturally occurring DNA derived from the proximal GC-box in the HSV TK promoter [53]. We found that the GC-box in the HSV TK promoter included a sequence (CGGCGCG) produced from two overlapping MLL1 morphemes: CGGCG and CGCG (Figure 4A). In transient expression assays, the GC-box recruited MLL1 to DNA to activate expression of a linked reporter gene [53]. This finding supports a role for MLL1-DNA interactions in activation of transcription. Furthermore, amino acid substitutions in the region encompassing the MT-domain abrogated transcription and reporter gene activation [53]. These findings support a role for interactions of the MT-domain with DNA in the regulation of transcription.

Results of another study provide evidence for functionality of MLL1 morphemes *in vivo*. Specifically, in an upstream promoter of the mouse *Hoxa9* gene, the study localized several CpG-rich clusters that were associated with MLL1 [54]. Gene-knockout experiments showed that MLL1 was required for protection of the CpG clusters from methylation [54]. We find that the CpG clusters in the *Hoxa9* promoter include MLL1 morphemes (Figure 4B). Isolated morphemes include CGCG, CGCCG, and CGCGCG. A MLL-protected cluster (CGGGCGGGCG) is produced from overlap of CGGGCG and CGGGCG. Thus, results of MLL-knockout experiments provide support for a role for MLL1 morphemes in an *in vivo* context.

### Morpheme occurrences in CpG islands

The finding that the MLL1 morphemes are CpG-rich raises the question of whether they are localized in CGIs in order to recruit MLL1 to chromatin. However, since the morphemes are relatively short *a priori* one could suspect that they may appear frequently in human genomic DNA just by chance: once every 256 bps for a 4-nucleotide motif; once every 1024 bps for a 5-nucleotide motif; once every 4094 bps for a 6-nucleotide motif. To examine this issue, we counted morpheme occurrences in total human genomic DNA. We find that morpheme frequencies in genomic DNA are relatively low. For example: CGCG (4 bps) occurs once per 53,977 bps; CGACG/CGTCG (5 bps) occurs once per 210,681 bps; and CGCGCG (6 bps) occurs once per 1,546,669 bps.

To evaluate more rigorously a possible connection between MLL1 morphemes and CGIs, we followed a previously described statistical model [49]. The statistical procedure partitions the human genome according to occurrences of a given MLL1 morpheme in CGIs and in regions outside CGIs. The probabilistic model assumes that the total genomic DNA is generated by a memoryless or Markov source. The statistical derivations are based on the principle of large deviations, often referred to as p-value analyses [56]. Results revealed that frequent morpheme occurrences in CGIs were statistically significant with β ≈ 10^-50^ (detailed in methods section).

To further assess a possible association of MLL1 morphemes with CGIs, we examined individual human chromosomes and total genomic DNA for morpheme occurrences. The analysis compared expected frequencies for random occurrences to observed morpheme frequencies in CGIs. We found that morpheme-occurrences in CGIs exceeded the values expected for random distribution in each human chromosome and in total genomic DNA (Additional file 3: Table S2).

For morpheme frequencies in CGIs, we noted the following trend: CGCG > CGCCG/ CGGCG > CGCCCG/ CGGGCG > CGCGCG > CGTCG/ CGACG > CGTGCG /CGCACG > CGTCCG/CGGACG > CGTACG. As expected, the frequencies are influenced by morpheme-length. Nonetheless, the trend indicates a bias in favor of GC-rich morphemes. For example, in CGIs, a 5-bp morpheme (CGCCG/ CGGCG) occurred 188,320 times while CGTCG/ CGACG occurred 38,647 times. In CGIs, a 6 bp morpheme (CGCCCG/ CGGGCG) occurred 62,702 times while CGTGCG /CGCACG occurred 18,184 times. Overall, the observed trend is consistent with a possible connection between MLL1 morphemes and CGIs since a high G + C content is a hallmark of sequences localized in CpG islands [5].

Additionally, we performed statistical evaluations of CpG-rich motifs that did not appear in results of SELEX assays. The analysis revealed that the non-motifs also are associated with CGIs. However, except for CGGCCG, the overall frequencies of non-motifs in CGIs were much lower than those observed for MLL1 morphemes (Additional file 4: Table S3). For example: in CGIs, CGAGCG/CGCTCG occurs 23,438 times; CGACCG/ CGGTCG occurs 10,009; CGAACG/CGTTCG occurs 5,019 times; CGATCG occurs 1,686 times.

### Occurrences of MLL1 morphemes in classified POLII promoters

Human POLII promoters can be classified into three groups: group I (about ~ 30%) does not have a CpG island at their TSS. Group II (about ~ 60%) has a single CpG island at their TSS. Group III (about ~ 10%) has two or more CpG islands in the vicinity of their TSSs [57]. Generally, the density of CpG dinucleotides in genomic DNA positively correlates with positions of H3K4me3 marks in chromatin, indicating that these two properties are mechanistically linked [57,58]. CpG-rich promoters may be enriched in RNA polymerase II poised for transcription [16]. In contrast, by default, AT-rich promoters are transcriptionally inactive (19).

**Figure 5:**
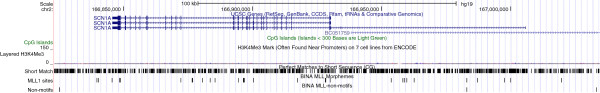
**Human genomic DNA without a CGI.** Track labeled “Short Match” marks the position of CpGs. Track-labeled “MLL1 sites” marks morpheme positions; “non-motifs” mark the position of sequences not found in results of SELEX assays [24]. Track labeled “Layered H3K4me3” shows the position of H3K4me3 marks.

Promoter classifications [57] led us to examine the distribution pattern of MLL1 morphemes in human genomic DNA with respect to CGI positions, overall CpG occurrences, and H3K4me3 modification patterns. We present three representative examples, chosen for comparison with results of POLII promoter classification [57]. The first example covers a region that does not include a CGI (Figure 5). The depicted segment is about 211,000 bp long. It includes a protein-coding gene (*SCN1A*), many CpGs but not a CGI. Figure 5 shows that MLL1 morphemes are scattered throughout the segment, possibly reflecting random occurrences (track labeled MLL1 sites). As observed previously [57], we did not find H3K4me3 marks for nucleosomes associated with that region in human genomic DNA (Figure 5, track labeled Layered H3K4me3).

**Figure 6:**
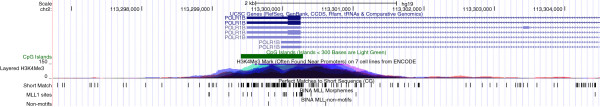
**Human genomic DNA with one CG encompassing *POLR1B *gene.** Horizontal green bar marks the CGI position. Track labeled “Short Match” marks the position of CpGs. Track-labeled “MLL1 sites” marks morpheme positions; “non-motifs” mark the position of sequences not found in results of SELEX assays [24]. Track labeled “Layered H3K4me3” shows the position of H3K4me3 marks, determined by the ENCODE project using a variety of cell lines including: lymphoblastoid cells (GM12878), H1 human embryonic stem cell line (H1-hESC), human skeletal muscle myoblasts (HSMM), human umbilical vein endothelial cells (HUVEC), erythroleukemia type cell line (K562), normal human epidermal keratinocytes (NHEK), and normal human lung fibroblasts (NHLF) [59]. A layered representation is displayed in order to provide an overview of H3K4me3 profiles [59].

The second example shows a region that includes a single CGI [57] encompassing TSSs of various *POLR1B* transcripts (Figure 6). Consistent with sequence characteristics of CGIs, frequency of CpG dinucleotides is relatively high within the island and tails into flanking sequences designated in literature as shores and shelves (Figure 5, lane labeled short match). The MLL1 morphemes are primarily localized within the island (Figure 6). Layers of H3K4me3 marks encompass the CpG island and extend into the island’s shores and shelves.

**Figure 7:**
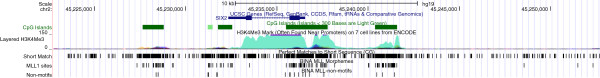
**Human genomic DNA with several CGIs and *SIX2 *gene.** Horizontal green bars mark the position of CGIs. Track labeled “Short Match” marks the position of CpGs. Track-labeled “MLL1 sites” marks morpheme positions; “non-motifs” mark the position of sequences not found in results of SELEX assays [24]. Track labeled “Layered H3K4me3” shows the position of H3K4me3 marks [59].

The third example shows a DNA segment that contains several CGIs and includes a region spanning a protein-coding gene known as *SIX2*[57]. Figure 7 shows that the MLL1 morphemes are densely packed within the islands. In contrast, the distribution of CpGs occurrences is significantly more broad and extends to surrounding shores and shelves of the CGIs. As noted previously [57], the H3K4me3 marks encompass the regions that includes a high CpG density. Since SIX2 functions in myogenesis [60], H3K4me3 marks are primarily observed for HSMM cells, human skeletal muscle myoblast (Figure 7).

### Morpheme occurrences in human *HOX* loci

In both *Drosophila* and vertebrates, the homeotic genes play essential roles in correct patterning of the body plan [61]. TrxG complexes and PcG complexes maintain the expression pattern of genes localized in appropriate domains [61,62]. As in mice, the human homeotic genes are organized into four clusters: *HOXA*, *HOXB*, *HOXC*, and *HOXD*[63]. This group of genes encode a family of transcription factors that play fundamental roles in morphogenesis during development [42]. Notably, several genes in the clusters include known MLL1 targets [42,47,55].

**Figure 8:**
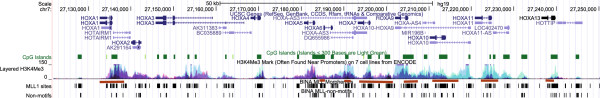
**Localization of CGIs and MLL1 morphemes in DNA segments encompassing human *HOXA *locus.** Horizontal green bars mark the position of CGIs. Horizontal brown bars mark MLL1 binding segments observed in ChIP assays [47]. Track-labeled “MLL1 sites” marks morpheme positions; “non-motifs” mark the position of sequences not found in results of SELEX assays [24]. Track-labeled “Layered H3K4me3” shows the position of H3K4me3 marks [59].

**Figure 9:**
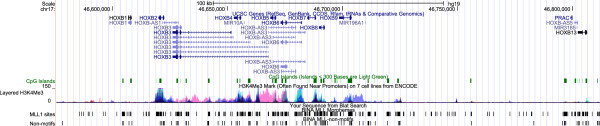
**Localization of CGI and MLL1 morphemes in DNA segments encompassing human *HOXB *locus.** Horizontal green bars mark the position of CGIs. Track labeled “MLL1 sites” marks the position of MLL1 morphemes; “non-motifs” mark the position of sequences not found in results of SELEX assays [24]. Track-labeled “Layered H3K4me3” shows the position of H3K4me3 marks [59].

**Figure 10:**
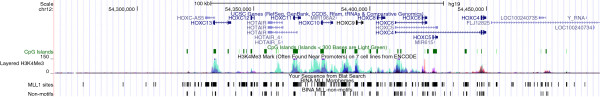
**Localization of CGI and MLL1 morphemes in DNA segments encompassing human *HOXC *locus.** Horizontal green bars mark the position of CGIs. Track-labeled “MLL1 sites” marks morpheme positions; “non-motifs” mark the position of sequences not found in results of SELEX assays [24]. Track-labeled “Layered H3K4me3” shows the position of H3K4me3 marks [59].

**Figure 11:**
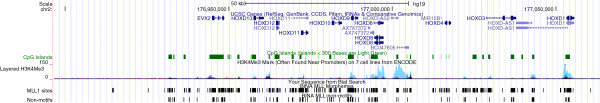
**Localization of CGI and MLL1 morphemes in DNA segments encompassing human *HOXD *locus.** Horizontal green bars mark the position of CGIs. Track-labeled “MLL1 sites” marks morpheme positions; “non-motifs” mark the position of sequences not found in results of SELEX assays [24]. Track-labeled “Layered H3K4me3” shows the position of H3K4me3 marks [59].

**Figure 12:**
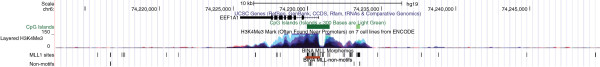
**Zoom-out view of MLL1 morpheme occurrences in *EE1F1A *locus.** Horizontal green bars mark the position of CGIs. Horizontal brown bar marks the chromatin segment bookmarked by MLL1 during mitosis [31]. Track-labeled “MLL1 sites” marks morpheme positions; “non-motifs” mark the position of sequences not found in results of SELEX assays [24]. Track labeled “Layered H3K4me3” shows the position of H3K4me3 marks [59].

Numerous CGIs are spread across the human *HOX* loci: about 31–36 CGIs/locus (Figures 8, 9, 10, 11 and 12). In these loci, the MLL1 morphemes are primarily localized in CGIs and in some cases tail into the shores and shelves of the islands (Figures 8, 9, 10, 11 and 12). A CGI may be associated with a bidirectional promoter regulating the expression of a *HOX* gene and a noncoding RNA. Examples include a CGI that include *HOXA1* and *HOTAIRM* promoters (Figure 8). Transcription of *HOTAIRM1* originates from the same CpG island that embeds the start site of *HOXA1*[64]. Similarly, a CGI encompasses a bidirectional promoter that regulates the expression of *HOXA13* and *HOTTIP* (Figure 8). Transcription of *HOTTIP* produces a noncoding RNA, implicated in maintaining active chromatin to coordinate the expression of genes in *HOXA* locus [65]. Transcription initiation site of another noncoding RNA gene (*HOXD-AS1*) is within a CGI that includes the coding region of *HOXD1* (Figure 11).

In the human *HOXA* locus, a previous study discovered extensive MLL1 binding events to a transcriptionally active chromatin domain [47]. In ChIP assays of a human monocytic cell-line (U937), MLL1 was localized to chromatin segments encompassing *HOXA1* and the 5′ *HOXA* subcluster including *HOXA7*, *HOXA9*, *HOXA10*, *HOXA11*, and *HOXA13* (Figure 8). Binding of MLL1 to these genes correlated with high-levels of their expression [47]. We find that MLL1 morphemes occur frequently in chromatin regions with which MLL1 associates (Figure 8).

We cover several examples illustrating the correspondence of morpheme occurrences to CGIs in human *HOXA* locus and to MLL1 associated regions determined by ChIP assays [47]. These regions are marked by horizontal brown-bars in Figure 8. ChIP assays localized an MLL-bound segment that included the TSS of *HOXA1*, extending into the transcribed region of the gene [47]. We find that the corresponding genomic DNA segment encompasses two CGIs that contain clusters of MLL1 morphemes (Figure 8). A short MLL-associated chromatin segment includes the *HOXA5* promoter and extends to the second exon in *HOXA6*[47]. The MLL-bound segment is within a CGI that contains two clusters of MLL1 morphemes (Figure 8). A long MLL-bound segment encompasses four CGIs that include several clusters of morpheme. A shorter MLL1 associated segment overlaps with a CGI that includes a cluster of morphemes; similarly sized MLL-bound segments also encompass CGIs that contain clusters of MLL1 morphemes (Figure 8).

In the human *HOXB* locus, CGIs are associated with promoters of several genes including *HOXB5*, *HOXB7*, and *HOXB9* (Figure 9). Intra- and inter-genic islands occur often and include both isolated and overlapping MLL1 morphemes (Figure 9). The *HOXC* locus primarily contains intra- and inter-genic CGIs (Figure 10). *HOXC8*, *HOXC9*, and *HOXC10* promoters are within CGIs that extend into coding sequences. These CGIs also include MLL1 morphemes (Figure 10). TSSs of genes in *HOXD* locus are often within CGIs that contain MLL1 morphemes (Figure 11). Examples include CGIs encompassing *HOXD1*, *HOXD6*, *HOXD8*, *HOXD9*, *HOXD12*, and *HOXD13* promoters (Figure 11).

### Morpheme occurrences in chromatin regions bookmarked by MLL1 during mitosis

To further assess the relevance of MLL1 morphemes to biological functions, we examined chromosomal regions reported to bind MLL1 during mitosis [31]. Binding of MLL1 to these regions preserved the memory of genes that were highly active prior to the onset of cell division [31]. The assays primarily focused on regions encompassing promoter sequences of POLII genes [31]. Therefore, we analyzed results of ChIP assays to determine whether the MLL-bound chromatin segments mapped to CGIs and to evaluate whether the bookmarked segments included MLL1 morphemes. We cover three representative examples, selected to compare our findings to figures discussed in a previous publication [31]. The first example deals with association of MLL1 with a chromatin segment that includes the TSS of *EEF1A1* gene. This association appeared exclusively in mitotic chromosomes [31]. The MLL-bound segment included the major TSS of *EEF1A1*, extending into the first exon and the first intron of the gene (brown-bars in Figure 12). We find that the corresponding genomic DNA is within a CGI and includes clusters of both isolated and overlapping MLL1 morphemes (Figure 12, track labeled MLL1 sites).

**Figure 13:**
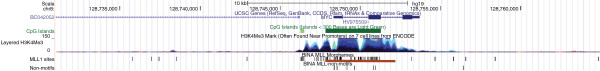
**Zoom-out view of morpheme occurrences in *MYC *locus.** Horizontal green bars mark the position of CGIs. Horizontal brown bars mark the chromatin segment bookmarked by MLL1 during mitosis [31]. Track labeled “MLL1 sites” marks the position of MLL1 morphemes; “non-motifs” mark the position of sequences not found in results of SELEX assays [24]. Track labeled “Layered H3K4me3” shows the position of H3K4me3 marks [59].

**Figure 14:**
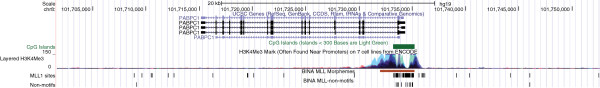
**Zoom-out view of morpheme occurrences in *PABPC1 *locus.** Horizontal green bar marks the position of CGIs. Horizontal brown bar marks the chromatin segment bookmarked by MLL1 during mitosis [31]. Track-labeled “MLL1 sites” marks morpheme positions; “non-motifs” mark the position of sequences not found in results of SELEX assays [24]. Track labeled “Layered H3K4me3” shows the position of H3K4me3 marks [59].

The second example covers the association of MLL1 with the *MYC* locus (Figure 13). ChIP assays revealed that MLL1 was preferentially bound to the *MYC* locus during mitosis whereas POLII occupied the locus in the interphase stage of cell-cycle [31]. In mitotic chromosomes, the MLL1-associated chromatin segment included sequences from ~0.5 kb upstream to ~1 kb downstream of the TSS of *MYC*[31]. The corresponding DNA segment encompasses a CGI that contains numerous clusters of MLL1 morphemes (Figure 13). *PABPC1* locus provides an example of numerous occurrences of both isolated and overlapping morphemes in a region bookmarked by MLL1 during mitosis [31]. The bookmarked segment (2.5 kb) is within a CGI that spans the promoter, the first exon, and part of the first intron of *PABPC1* gene (Figure 14). MLL1 morphemes are spread across the DNA segment localized in ChIP assays (Figure 14). The segment includes several morpheme-overlaps produced from permutations of CGCG, CGCCG, CGCCCG, and complements of these sequences. Examples include CGCGCCG, CGCCGCG, CGCGGGCG, CGCGGCG, CGCGGCG in PABPC1 promoter. Notably, the *PABPC1* associated CGI contains multiple occurrences of morphemes that also occur in a region that *in vivo* MLL1 protected from CpG methylation (Figure 4B).

### On gene bookmarking during mitosis

Overall, results of our analyses implied that interactions of MLL1 with its morphemes may contribute to gene bookmarking events that preserved the memory of genes that were highly active prior to mitosis [31]. However, evidence is lacking for involvement of other MLL1 family members in gene bookmarking events. As MLL1, MLL2/KMT2B binds non-methylated CpGs [25]. Furthermore, a study has shown binding of MLL2 to a POLII promoter within a CpG island [66]. However, ChIP assays revealed that MLL2 was evicted from mitotic chromatin indicating that MLL2 did not contribute to gene bookmarking during mitosis [31]. The structure of the other two family members (MLL3/ KMT2C and MLL4/ KMT2D) does not contain an MT-domain. Therefore, it seems unlikely that MLL3 and MLL4 would interact with CpG rich motifs localized in CpG islands.

MLL1 is a component of relatively large and dynamic multiprotein complexes [13]. Therefore, one may ask whether other components in these complexes would contribute to gene-bookmarking by MLL1 [31]. In protein networks, MLL1 interacts with several proteins, including MEN1, RBBP5, and ASH2L [67], (Figure 1). All three proteins associate with MLL1 during both interphase and mitosis [31]. In MLL-deficient cells, most of RbBP5, ASH2L, and MEN1 were localized to the cytoplasm, indicating that their association with mitotic chromatin was MLL-dependent [31]. Even though MEN1 interacts with DNA, the binding is not DNA-sequence-specific [68]. MEN1 also associates with a variety of DNA structures, including Y-structures, branched structures, and 4-way junction structures [68]. In literature surveys, we did not find evidence for direct interactions of MEN1 with CpG-containing sequences. Furthermore, while during mitotic silencing of highly expressed genes MEN1 was associated with mitotic chromatin, MLL1 was required for this association [31].

Other candidates for gene-bookmarking include LEDGF/p75 (Figure 1). LEDGF is best known for its role in tethering to chromatin protein-complexes that integrate the HIV-1 genome into the host-cell chromosomes [69]. LEDGF primarily associates with chromatin in regions downstream of TSSs, to effect gene-specific HIV-1 integration [70]. Furthermore, in contrast to MLL1 [24,53], LEDGF does not bind CpG-rich DNA sequences.

Since MLL1 is best known for its H3K4 methyltransferase activity, one may expect that gene bookmarking by MLL1 could involve mechanisms dealing with trimethylation of histone H3 [31]. However, MLL1 was dispensable for preserving histone H3K4 methylation during mitosis, indicating that MLL1 served H3K4 methyltransferase-independent functions to propagate active chromatin during mitosis [31]. Furthermore, during mitosis, SETD1A was evicted from mitotic chromosomes, implying that it did not contribute to gene-bookmarking events [31]. SETD1A is the major H3K4 methyltransferase during the interphase and targets many nucleosome-associated genes for histone H3 modifications [17]. Both SETD1A and SETD1B interact with a protein (CXXC1/Cfp1) that binds unmethylated CpG [26,43], Figure 1. Earlier genome-wide studies localized CXXC1 to CGIs and deduced that CXXC1 functions included recruitment of SETD1A and SETD1B to chromatin for trimethylation of H3K4 [71]. However, subsequent studies revealed that while CXXC1 played a key role in organizing genome-wide H3K4me3 in mouse ES cells, its DNA binding domain was not required for recruitment of enzymes that produced H3K4me3 marks on CGI-associated nucleosomes [72]. While CXXC1 is crucial for early embryonic development and regulates genomic cytosine methylation patterns [73-75], it remains to be determined whether CXXC1 may also play a role in gene bookmarking during mitosis.

### Occurrences of overlapping MLL1 morphemes

We noted that in some cases, morphemes overlapped in various orders and combinations. Based on statistical criteria (described in the Methods section), occurrences of morpheme overlaps in CGIs are even rarer events than those obtained for isolated morphemes. We found that morpheme overlaps creating long sequences appeared infrequently in human genomic DNA. We noticed that morpheme overlaps could be produced from a repeated DNA sequence element. Examples include CGG repeats associated with genetic abnormalities. (CGG)_n_ creates morpheme overlaps of the following form: CGGCGGCGGCGGCGGCGG etc.

Notable examples include the *FMR1* locus in which CGG expansion causes mental retardation [76]. This expansion arises in a CGI associated with Fragile X Syndrome, in the 5′ untranslated region of the *FMR1* gene [77]. In normal individuals, repeat-size varies from 6 to 54 CGG [78]. All alleles with greater than 52 repeats, including those identified in a normal family, are mitotically unstable [78]. Remarkably, in carriers *FMR1* transcription increases, displaying a positive correlation between repeat number and levels of *FMR1* transcripts [79]. Additionally, carriers display changes in TSS utilization [76]. Thus, repeated overlapping morphemes, downstream of transcription initiation sites, may influence TSS utilization and upregulation of gene expression.

Several overlapping MLL1 morphemes are dispersed across the human *HOX* loci (Figures 8, 9, 10 and 11). In some cases morpheme overlaps are localized upstream or near TSS of specific genes. Examples include morpheme overlaps in promoter/upstream sequences of genes in various loci: CGCGCGCGCG, *HOXA4*; CGCCCGCCCGCCGCCCGCCCG *HOXA6*; CGCCCGCGCCCGGCG, *HOXA7*; CGGCGCGCGCG, *HOXA11*; two repeats (CGCCGCCGCCGCCGCCGCCGCCCG and CGCCGCCCGCCGCCGCCGCCG), *HOXC8*; and CGGCGGCGGCGGCG, *HOXD10*.

In some cases, overlapping morphemes are localized in coding regions producing repeated amino acid residues in a polypeptide chain. Notable examples include morpheme overlaps in *HOXA13* and *HOXD13* coding sequences producing tracts of alanines. Amplification of DNA sequences in a *HOXD13* exon causes Syndactyly, fusion of digits in fingers [63]. It is thought that Syndactyly is due to expansion of alanine-tracts [63]. However, it seems plausible that overlapping morphemes in coding sequences may play a regulatory role at the level of gene expression. In fact, an emerging view is that gene regulatory and coding sequences are more intermingled than once believed [80].

Statistical criteria indicate that morpheme overlaps are rare events in genomic DNA, raising the question of whether occurrences of overlapping morphemes could play a role in cellular functions regulated by MLL1. In this context, we noted that a previous study found that the SET domain in MLL1 self-associated to form homo-oligomeric complexes [81]. This association was observed in various experimental settings including yeast two-hybrid methodology, biochemical studies, and deletion analyses [81]. The study found a similar self-association for the SET domain in the *Drosophila* trithorax [81].

In leukemogenic MLL1 fusion proteins, the SET domain is deleted and replaced with over 40 different translocation partners [20]. Invariably, the MLL1 MT-domain is retained at the amino-terminus of fusion proteins [20]. MLL1 fusion partners include transcriptional activators that upregulate gene expression in leukemic cells. Also, there are partners that impart transcriptional activating properties to MLL1 fusion proteins by promoting dimerization [82]. Dimerization of fusion proteins immortalized hematopoietic cells by upregulating transcription of several endogenous genes [82]. Interestingly, protein-dimerization enhanced the binding of MLL1 amino-terminus to regulatory regions leading to upregulation of linked genes [82].

Since normal forms of MLL1 self-associate *via* the SET domain [81] to produces homo-oligomeric complexes [81], it seems plausible that as observed for leukemic cells [82], association of MLL1 molecules could enhance the affinity of MLL1 for DNA. Furthermore, self-association might operate in linking MLL1 molecules so that they would reside simultaneously on different maintenance elements in chromosomes [81]. This mechanism would integrate the activity of MLL1 in activation of a target gene, shared target genes, or both [81]. Propagation of MLL1 association with DNA may arise from a combination of two molecular events: binding of MLL1 to overlapping morphemes and MLL1 oligomerization *via* the SET domain. Cooperative DNA binding, *via* self-association, often increases the DNA binding specificity of a protein. We imagine that overlapping MLL1 morphemes may facilitate MLL1 self-association linking MLL1 molecules to reside cooperatively on DNA sequence elements (TREs) that maintain cellular memory during development. Our data indicate that such TREs also could function in gene-bookmarking to preserve the memory of highly active genes during mitosis. Also, one could imagine that overlapping morpheme occurrences may facilitate localized propagation of MLL1 binding to DNA to maintain a nucleosome-free region and, thus, an open chromatin configuration.

## Conclusions

Annotation of the human genome has involved numerous experimental and computational strategies to identify and describe DNA sequences that are important to cellular functions. However, despite cutting-edge advances, we lack a complete understanding of the function of CpG islands, which were discovered some time ago [4,5]. Results of our analysis provide suggestive evidence for specific sequence motifs in CGIs that may function in the recruitment of MLL1 to mitotic chromatin. We show that various combinations of MLL1 morphemes occur in chromatin regions bookmarked by MLL1 during mitosis [31]. Thus, our results implicate the MLL1 morphemes in sequence-features that define the mammalian TREs. Our results also suggest a role for overlapping morphemes in producing multiple MLL1 binding events, linking MLL1 molecules so that they would reside simultaneously on different maintenance elements in chromosomes, as previously proposed [81].

Our findings also may explain why CGIs often extend to include promoter, exonic, and intronic sequences of genes. By binding CGIs, MLL1 might preserve and maintain an open chromatin configuration to regulate gene expression and to facilitate rapid gene activation upon mitotic exit. Association of MLL1 with CGIs agrees with a global role for MLL1 in regulation of transcription [47].

Apparently, our findings provide the first evidence for the existence of potential TREs in mammalian genomic DNA and the first evidence for a connection between CGIs and gene-bookmarking by MLL1 to transmit the memory of highly active chromatin states during cell-division. Because of the strong connection of TREs and PREs in *Drosophila*[32], we speculate that the MLL1 morphemes may play a dual role: (1) contribute directly to the recruitment of mammalian TrxG complexes to chromatin and (2) contribute indirectly, or directly, to the recruitment of PRC2 complexes to chromatin to repress transcription. This possibility is consistent with the finding that the mammalian PRC2 repressive complex binds CGIs [34] and our discovery of frequent occurrences of MLL1 morphemes in CpG islands.

## Methods

### Identification of MLL1 morphemes and their localization in human genomic DNA

We identified the MLL1 binding units by analyzing results of reported SELEX- and PCR-based assays. These assays were conducted to determine the DNA binding properties of the MLL1 MT-domain [24]. In our analyses, we included counting schemes to assess the number of CpGs and to identify nucleotides that appeared between CpG dinucleotides in each cloned inserts.

To count genomic occurrences of MLL1 morphemes, we downloaded nucleotide sequences of CGIs and human chromosomes from the human genome browser at UCSC [83]. A Perl script was written to determine occurrences of each morpheme in downloaded sequences and to create outputs displaying the results. We followed various counting schemes. We found including or omitting morpheme overlaps gave about the same number of counts (variation among procedures was less than 10%).

To localize genomic positions of MLL1 morphemes, we retrieved genomic DNA (Hg19) from the Genome Browser at UCSC [83]. Sequence analyses involved scanning the human genome for morpheme occurrences, using Perl scripts [50]. Similarly, we developed script to create outputs (bed files) to display the position of MLL1 morphemes on the Genome Browser at UCSC [50]. Tools offered by the browser facilitated examining genomic maps in context of landmarks, including the position of genes, CGIs, and chromatin modification patterns [83,84].

### Studies of promoter sequences of human genes

To analyze promoter sequences of POLII genes, we obtained the accession number of human cDNAs from the UCSC database [83]. Heather Trumbower (at UCSC) wrote queries and retrieved the accession number of 44,338 cDNAs, organized according to their position in human chromosomes. To reduce sequence-redundancy, we selected one cDNA per gene. Subsequently, we computationally removed cDNAs that appeared to be incomplete. Accession numbers of remaining cDNAs were uploaded on the table browser at UCSC to obtain the nucleotide sequence of corresponding promoters: -500 to transcription start site. Since the human genome may contain multiple copies of a given gene [85], we chose one promoter to represent redundant genes.

Afterwards, we followed previously described methods [49,50,86] to create a database for retrieving information about the final set (15,906) of promoters. The database (RF_data_06) tracked the number of occurrences as well as the position of all possible 9-mers in POLII promoters, with respect to TSSs. For statistical evaluations, the database included counts of 9-mers in total human genomic DNA and in repetitive DNA sequences [49]. For promoter analyses, we queried RF_data_06 to obtain counts for a given subsequence (i.e. CGCG, CGNCG, CGNNCG, and MLL1 morphemes) at each nucleotide position (-500 to -1).

### Statistical evaluation

For statistical evaluation, we followed a previously described approach [49]. Briefly, Regnier and Szpankowski have shown that occurrences of words in a randomly generated text (based on either Bernoulli or Markov model) are normally distributed around a mean [87]. We used their findings to perform statistical derivations based on the principle of large deviations [49].

We chose the following notations: L_*G*_ length of total genomic DNA, L_*E*_ total length of CGIs, and L_*F*_ length of regions that do not correspond to CGIs. Thus, L_F_ = L_G_ - L_E_

Subsequently, we created a motif table (*w*_1_, …, *w*_*M*_), consisting of MLL1 morphemes to identify elements that matched sequences in L_*E*_ and L_*G*_

For 1 ≤ i ≤ M, we denote by E_i_, F_i_, and G_i_, respectively, the frequency of the i^th^ element (w_i_) in L_*E*_, L_*F*_ , and L_*G*_

Since L_E_ is significantly shorter than L_G_ as an approximation we assume |L_E_| < |L_F_| ≈ |L_G_|

Quantities of interest are total counts normalized with respect to length of analyzed sequences:

$$e_{i}=\frac{E_{i}}{L_{E}}$$

$$f_{i}=\frac{F_{i}}{L_{F}}$$

$$g_{i}=\frac{G_{i}}{L_{G}}$$

We made two additional justifiable approximations: f_i_ ≈ g_i_ and f_i_ ≈ p(w_i_)

Since |L_G_| is very large, within the margin of error, f_i_ approximates the probability of occurrence of morpheme w_i_ in genomic DNA.

As previously [49], we aimed to determine a threshold α_th_ so that we could assign statistical significance to cases in which e_i_ > αf_i_ (or e_i_ > αg_i_). Evaluations require comparing empirical data to a reference model. For reference, we chose a probabilistic model assuming that the genome is generated by a memoryless or Markov source. In this model, e_i_ and f_i_ become random variables.

As detailed above, we simplified the analysis by assuming that f_i_ = p(w_i_) is a constant. Subsequently, we determine whether for a given β, the event e_i_ > αf_i_ is *statistically significant* provided that the probability of e_i_ > αf_i_ is smaller than β. That is, P(e_i_ > αf_i_) < β (the chance of randomness that generates the event e_i_ > αf_i_ is very small). We set β = 10^-50^ to compute the α_th_ threshold.

From [87], we knew that Ei values should be normally distributed around a mean

$$E\left[E_{i}\right]=L_{E}p\left(w_{i}\right)$$

When E_i_ does not deviate more than $O\left(\sqrt{L_{E}p\left(w_{i}\right)}\right)$

$$E_{i}\sim N\left(L_{E}p\left(w_{i}\right),L_{E}\sigma^{2}\left(w_{i}\right)\right)$$

Where, N(μ, σ^2^) denotes the normal distribution with mean μ and variance σ^2^

When E_i_ deviates from $O\left(\sqrt{L_{E}p\left(w_{i}\right)}\right)$ another probabilistic law would govern the Ei behavior: namely, the large deviations law [56]. Previously Regnier and Szpankowski [87] proved

(1) $$p\left(\mathrm{Ei}<\left(1+\delta \right)L_{E}p\left(w_{i}\right)\right)<\frac{1}{\sqrt{2\pi L_{E}}}\exp\left(-L_{E}I\left(\delta \right)\right)$$

Where, I(δ) is a complicated function of δ that depends on moment generation functions [88]. To compute threshold α = 1 + δ > 1, we estimate δ from

$$P\left(e_{i}<\left(1+\delta \right)p\left(w_{i}\right)\right)<\beta$$

That equation translates into P(E_i_ > (1 + δ)L_E_p(w_i_)) < β which is clearly within the large deviations domain.

For the analyses, we need to apply Eq. (1). However, numerical computations of the large deviation function I(δ) are rather cumbersome. Therefore, we followed approximations, noting that a good bound was needed only for the large deviation probability. Ignoring overlapping morphemes, E_i_ would be a sum of Bernoulli independent random variables. If that case, the following bound can be found (cf. for example, Ref. [88]):

(2) $$P\left(E_{i}>\left(1+\delta \right)L_{E}p\left(w_{i}\right)\right)<\exp\left(-L_{E}I\left(\delta^{2}/3\right)\right)$$

To be rigorous and take into account overlapping morphemes, we must somewhat relax equation (2). Referring to Azuma’s inequality (cf. Ref. [56]), we obtain:

(3) $$P\left(E_{i}>\left(1+\delta \right)L_{E}p\left(w_{i}\right)\right)\leq \exp\left(-L_{E}p\left(w_{i}\right)\delta^{2}/2\right)$$

From equation (2) and (3), we obtain the following estimate for threshold α_th_ = 1 + δ

$$1+\sqrt{\frac{2\ln\beta^{-1}}{L_{E}p\left(w_{i}\right)}}\leq \;\alpha_{\mathrm{th}}\;\leq 1+\sqrt{\frac{3\ln\beta^{-1}}{L_{E}p\left(w_{i}\right)}}$$

## Abbreviations

CGIs: CpG islands; H3K4: Lysine 4 on histone H3; PcG: Polycomb group; POLII: RNA polymerase II; PRC1: Polycomb repressive complexes 1; PRC2: Polycomb repressive complexes 2; PREs: Polycomb response elements; TREs: Trithorax response elements; TSSs: Transcription start sites; TrxG: Trithorax group; UCSC: University of California Santa Cruz.

## Competing interests

The authors declare that they have no competing interests.

## Authors’ contributions

MB designed experiments, performed analyses, and wrote the manuscript. PW wrote programs for creating the database and performed statistical evaluations. EN analyzed results of SELEX assays and identified the MLL1 morphemes. NZ mapped the position of MLL1 morphemes in human genomic DNA. JX improved the programs used in mapping studies. RP and ZG analyzed a listing of human protein-coding-genes downloaded from the human genome browser at UCSC. MF created a database of 9-mers for studies of promoter regions of human genes. BF contributed to statistical evaluations. DW wrote programs to map the position of MLL1 morphemes in human chromosomes. All authors read and approved the final manuscript.

## Supplementary Material

Additional file 1: Figure S1 Occurrences of CpG-rich motifs in promoter regions of human protein-coding genes. Full magenta-circles correspond to CGNNCG, blue-circles to CGNCG, and empty red-circles to CGCG. Motif frequencies are shown as the function of nucleotide positions in promoter sequences, numbered with respect to TSSs. Click here for file

Additional file 2: Table S1 Frequency of morphemes and non-motifs in promoter sequences of POLII genes. Click here for file

Additional file 3: Table S2 Counts of expected and observed morpheme occurrences in CpG islands. Click here for file

Additional file 4: Table S3 Counts of expected and observed non-motif occurrences in CpG islands. Click here for file
