# Supplementary figures and images for: Discovery of MLL1 binding units, their localization to CpG Islands, and their potential function in mitotic chromatin
Source: BMC Genomics. 2013 Dec 28;14:927. doi: 10.1186/1471-2164-14-927 (PMC3890651; doi:10.1186/1471-2164-14-927)

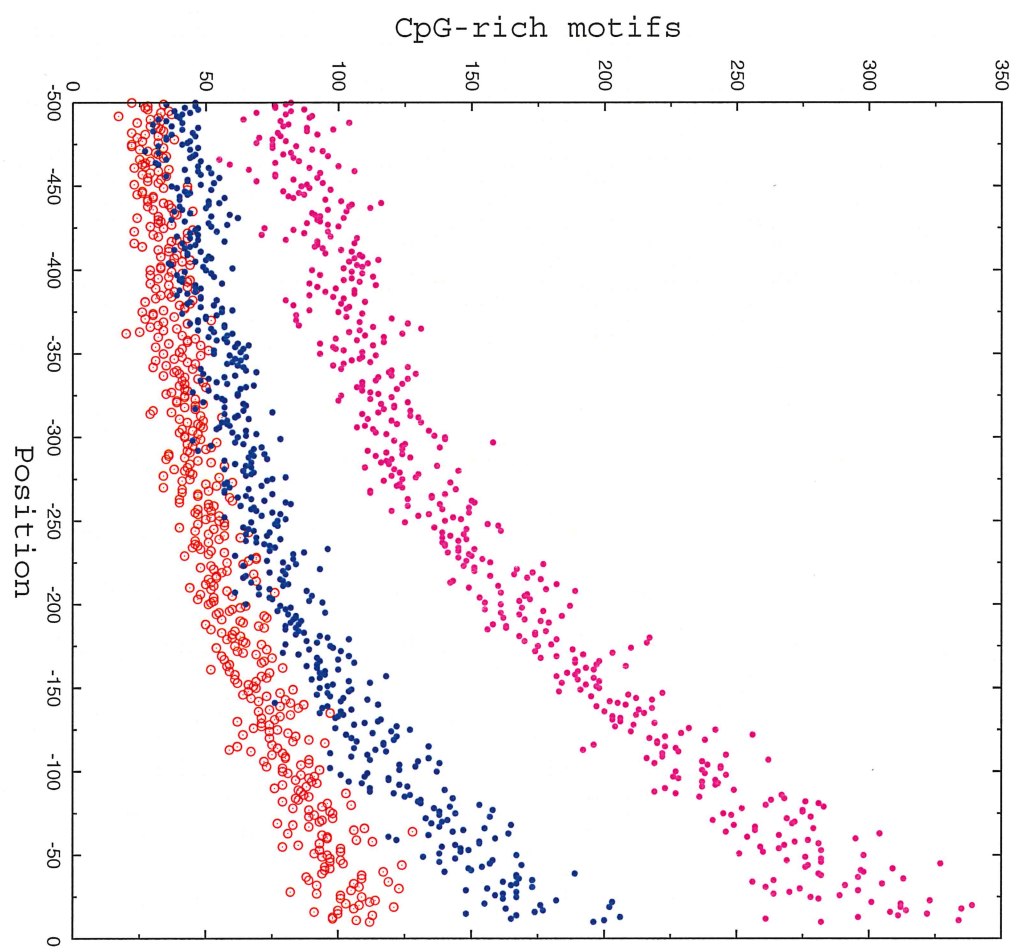

Supplement: Additional file 1: Figure S1 — Occurrences of CpG-rich motifs in promoter regions of human protein-coding genes. Full magenta-circles correspond to CGNNCG, blue-circles to CGNCG, and empty red-circles to CGCG. Motif frequencies are shown as the function of nucleotide positions in promoter sequences, numbered with respect to TSSs. [file 1471-2164-14-927-S1.pdf]
