# Supplementary material for: Discovery of MLL1 binding units, their localization to CpG Islands, and their potential function in mitotic chromatin
Source: BMC Genomics. 2013 Dec 28;14:927. doi: 10.1186/1471-2164-14-927 (PMC3890651; doi:10.1186/1471-2164-14-927)
Supplement: Additional file 2: Table S1 — Frequency of morphemes and non-motifs in promoter sequences of POLII genes. [file 1471-2164-14-927-S2.pdf]

Supplemental Table 1

Frequency of morphemes and non-motifs in promoter sequences of POLII genes

Morpheme/compl	Promoter/counts
CGCG	27964
CGACG	13178
CGGCG	17440
CGTGCG	3487
CGCCCG	13086
CGGACG	6589
CGCGCG	3588
CGTACG	279

Non-motif/compl	Promoter/counts
CGAACG	665
CGACCG	1823
CGAGCG	4535
CGATCG	568
CGGCCG	10703
