# Supplementary material for: Discovery of MLL1 binding units, their localization to CpG Islands, and their potential function in mitotic chromatin
Source: BMC Genomics. 2013 Dec 28;14:927. doi: 10.1186/1471-2164-14-927 (PMC3890651; doi:10.1186/1471-2164-14-927)
Supplement: Additional file 3: Table S2 — Counts of expected and observed morpheme occurrences in CpG islands. [file 1471-2164-14-927-S3.pdf]

Supplemental Table 2

CHR1 to CHRY	Morpheme	Compl	CGIs, expected counts for random occurrences	CGIs, Observed counts
CHR1	CGCG		278	17118
CHR1	CGTCG	CGACG	68	3390
CHR1	CGCCG	CGGCG	130	17019
CHR1	CGCGCG		10	2231
CHR1	CGTGCG	CGCACG	36	1574
CHR1	CGCCCG	CGGGCG	205	5888
CHR1	CGTCCG	CGGACG	32	1384
CHR1	CGTACG		6	106
CHR2	CGCG		183	13437
CHR2	CGTCG	CGACG	49	2481
CHR2	CGCCG	CGGCG	86	13334
CHR2	CGCGCG		6	1703
CHR2	CGTGCG	CGCACG	25	1115
CHR2	CGCCCG	CGGGCG	128	4289
CHR2	CGTCCG	CGGACG	21	1033
CHR2	CGTACG		5	78
CHR3	CGCG		105	8348
CHR3	CGTCG	CGACG	26	1630
CHR3	CGCCG	CGGCG	45	8579
CHR3	CGCGCG		4	1143
CHR3	CGTGCG	CGCACG	13	824
CHR3	CGCCCG	CGGGCG	80	2767
CHR3	CGTCCG	CGGACG	12	703
CHR3	CGTACG		2	60
CHR4	CGCG		94	7775
CHR4	CGTCG	CGACG	25	1469
CHR4	CGCCG	CGGCG	42	7818
CHR4	CGCGCG		3	1020
CHR4	CGTGCG	CGCACG	13	714
CHR4	CGCCCG	CGGGCG	67	2419
CHR4	CGTCCG	CGGACG	11	751
CHR4	CGTACG		2	29
CHR5	CGCG		119	8930
CHR5	CGTCG	CGACG	31	1735
CHR5	CGCCG	CGGCG	52	8642
CHR5	CGCGCG		4	1089
CHR5	CGTGCG	CGCACG	15	758
CHR5	CGCCCG	CGGGCG	85	2807
CHR5	CGTCCG	CGGACG	13	752
CHR5	CGTACG		3	79
CHR6	CGCG		128	8729
CHR6	CGTCG	CGACG	32	1700

CHR6	CGCCG	CGGCG	57	8513
CHR6	CGCGCG		5	1097
CHR6	CGTGCG	CGCACG	16	789
CHR6	CGCCCG	CGGGCG	88	2663
CHR6	CGTCCG	CGGACG	14	660
CHR6	CGTACG		3	39
CHR7	CGCG		189	10521
CHR7	CGTCG	CGACG	49	1932
CHR7	CGCCG	CGGCG	89	10130
CHR7	CGCGCG		7	1372
CHR7	CGTGCG	CGCACG	25	1014
CHR7	CGCCCG	CGGGCG	130	3351
CHR7	CGTCCG	CGGACG	21	878
CHR7	CGTACG		4	70
CHR8	CGCG		113	7820
CHR8	CGTCG	CGACG	34	1528
CHR8	CGCCG	CGGCG	54	7571
CHR8	CGCGCG		5	1257
CHR8	CGTGCG	CGCACG	17	773
CHR8	CGCCCG	CGGGCG	73	2502
CHR8	CGTCCG	CGGACG	13	611
CHR8	CGTACG		3	40
CHR9	CGCG		137	8792
CHR9	CGTCG	CGACG	34	1705
CHR9	CGCCG	CGGCG	68	8624
CHR9	CGCGCG		4	1157
CHR9	CGTGCG	CGCACG	17	768
CHR9	CGCCCG	CGGGCG	99	2947
CHR9	CGTCCG	CGGACG	15	740
CHR9	CGTACG		3	46
CHR10	CGCG		153	8912
CHR10	CGTCG	CGACG	41	1641
CHR10	CGCCG	CGGCG	71	8653
CHR10	CGCGCG		5	1186
CHR10	CGTGCG	CGCACG	22	795
CHR10	CGCCCG	CGGGCG	99	2846
CHR10	CGTCCG	CGGACG	17	690
CHR10	CGTACG		4	42
CHR11	CGCG		154	9312
CHR11	CGTCG	CGACG	41	1885
CHR11	CGCCG	CGGCG	77	8956
CHR11	CGCGCG		5	1207
CHR11	CGTGCG	CGCACG	22	931
CHR11	CGCCCG	CGGGCG	106	3084
CHR11	CGTCCG	CGGACG	17	827
CHR11	CGTACG		4	61
CHR12	CGCG		138	7440

CHR12	CGTCG	CGACG	33	1513
CHR12	CGCCG	CGGCG	62	7682
CHR12	CGCGCG		5	985
CHR12	CGTGCG	CGCACG	18	687
CHR12	CGCCCG	CGGGCG	100	2503
CHR12	CGTCCG	CGGACG	15	620
CHR12	CGTACG		3	54
CHR13	CGCG		48	4470
CHR13	CGTCG	CGACG	13	777
CHR13	CGCCG	CGGCG	21	4215
CHR13	CGCGCG		2	528
CHR13	CGTGCG	CGCACG	6	371
CHR13	CGCCCG	CGGGCG	33	1302
CHR13	CGTCCG	CGGACG	5	338
CHR13	CGTACG		1	31
CHR14	CGCG		81	5938
CHR14	CGTCG	CGACG	21	1233
CHR14	CGCCG	CGGCG	38	5859
CHR14	CGCGCG		3	752
CHR14	CGTGCG	CGCACG	11	501
CHR14	CGCCCG	CGGGCG	57	1997
CHR14	CGTCCG	CGGACG	9	445
CHR14	CGTACG		2	31
CHR15	CGCG		93	6500
CHR15	CGTCG	CGACG	22	1333
CHR15	CGCCG	CGGCG	42	6467
CHR15	CGCGCG		3	839
CHR15	CGTGCG	CGCACG	12	553
CHR15	CGCCCG	CGGGCG	67	2211
CHR15	CGTCCG	CGGACG	10	523
CHR15	CGTACG		2	45
CHR16	CGCG		238	9317
CHR16	CGTCG	CGACG	63	1974
CHR16	CGCCG	CGGCG	127	9193
CHR16	CGCGCG		8	1110
CHR16	CGTGCG	CGCACG	34	1033
CHR16	CGCCCG	CGGGCG	157	3135
CHR16	CGTCCG	CGGACG	28	869
CHR16	CGTACG		5	52
CHR17	CGCG		361	11275
CHR17	CGTCG	CGACG	81	2352
CHR17	CGCCG	CGGCG	173	11108
CHR17	CGCGCG		11	1414
CHR17	CGTGCG	CGCACG	48	1078
CHR17	CGCCCG	CGGGCG	266	3875
CHR17	CGTCCG	CGGACG	37	967
CHR17	CGTACG		7	66

CHR18	CGCG		58	4143
CHR18	CGTCG	CGACG	16	814
CHR18	CGCCG	CGGCG	27	4036
CHR18	CGCGCG		2	595
CHR18	CGTGCG	CGCACG	8	421
CHR18	CGCCCG	CGGGCG	37	1275
CHR18	CGTCCG	CGGACG	6	350
CHR18	CGTACG		2	21
CHR19	CGCG		645	13264
CHR19	CGTCG	CGACG	135	3068
CHR19	CGCCG	CGGCG	326	12190
CHR19	CGCGCG		23	1655
CHR19	CGTGCG	CGCACG	77	1551
CHR19	CGCCCG	CGGGCG	481	4232
CHR19	CGTCCG	CGGACG	66	1139
CHR19	CGTACG		9	93
CHR20	CGCG		117	5663
CHR20	CGTCG	CGACG	32	1309
CHR20	CGCCG	CGGCG	58	5626
CHR20	CGCGCG		4	770
CHR20	CGTGCG	CGCACG	17	557
CHR20	CGCCCG	CGGGCG	75	1940
CHR20	CGTCCG	CGGACG	14	443
CHR20	CGTACG		2	28
CHR21	CGCG		37	2493
CHR21	CGTCG	CGACG	10	445
CHR21	CGCCG	CGGCG	19	2269
CHR21	CGCGCG		1	323
CHR21	CGTGCG	CGCACG	5	233
CHR21	CGCCCG	CGGGCG	23	744
CHR21	CGTCCG	CGGACG	4	226
CHR21	CGTACG		1	7
CHR22	CGCG		127	5479
CHR22	CGTCG	CGACG	32	1121
CHR22	CGCCG	CGGCG	66	5370
CHR22	CGCGCG		4	704
CHR22	CGTGCG	CGCACG	17	462
CHR22	CGCCCG	CGGGCG	84	1948
CHR22	CGTCCG	CGGACG	14	475
CHR22	CGTACG		2	22
CHRX	CGCG		83	5690
CHRX	CGTCG	CGACG	23	1425
CHRX	CGCCG	CGGCG	35	5848
CHRX	CGCGCG		3	714
CHRX	CGTGCG	CGCACG	12	594
CHRX	CGCCCG	CGGGCG	64	1787
CHRX	CGTCCG	CGGACG	9	515

CHRX	CGTACG		2	42
CHRY	CGCG		6	730
CHRY	CGTCG	CGACG	2	187
CHRY	CGCCG	CGGCG	3	618
CHRY	CGCGCG		0	91
CHRY	CGTGCG	CGCACG	1	88
CHRY	CGCCCG	CGGGCG	5	190
CHRY	CGTCCG	CGGACG	1	70
CHRY	CGTACG		0	16
TOTAL	CGCG		3139	192096
TOTAL	CGTCG	CGACG	804	38647
TOTAL	CGCCG	CGGCG	1477	188320
TOTAL	CGCGCG		110	24942
TOTAL	CGTGCG	CGCACG	420	18184
TOTAL	CGCCCG	CGGGCG	2208	62702
TOTAL	CGTCCG	CGGACG	349	16009
TOTAL	CGTACG		72	1158
