# Supplementary material for: Discovery of MLL1 binding units, their localization to CpG Islands, and their potential function in mitotic chromatin
Source: BMC Genomics. 2013 Dec 28;14:927. doi: 10.1186/1471-2164-14-927 (PMC3890651; doi:10.1186/1471-2164-14-927)
Supplement: Additional file 4: Table S3 — Counts of expected and observed non-motif occurrences in CpG islands. [file 1471-2164-14-927-S4.pdf]

Supplemental Table 3

#CHR1 to CHRY	non motif	compl	CGIs, expected counts for random occurrences	CGIs, Observed counts
CHR1	CGAACG	CGTTCG	15	480
CHR1	CGATCG		6	163
CHR1	CGACCG	CGGTCG	14	944
CHR1	CGAGCG	CGCTCG	27	2160
CHR1	CGGCCG		37	2808
CHR2	CGAACG	CGTTCG	11	321
CHR2	CGATCG		4	107
CHR2	CGACCG	CGGTCG	10	660
CHR2	CGAGCG	CGCTCG	17	1633
CHR2	CGGCCG		24	2112
CHR3	CGAACG	CGTTCG	6	248
CHR3	CGATCG		3	77
CHR3	CGACCG	CGGTCG	6	475
CHR3	CGAGCG	CGCTCG	10	1087
CHR3	CGGCCG		14	1369
CHR4	CGAACG	CGTTCG	6	175
CHR4	CGATCG		3	50
CHR4	CGACCG	CGGTCG	5	388
CHR4	CGAGCG	CGCTCG	9	911
CHR4	CGGCCG		11	1172
CHR5	CGAACG	CGTTCG	7	248
CHR5	CGATCG		3	84
CHR5	CGACCG	CGGTCG	6	469
CHR5	CGAGCG	CGCTCG	11	1097
CHR5	CGGCCG		15	1322
CHR6	CGAACG	CGTTCG	7	258
CHR6	CGATCG		3	67
CHR6	CGACCG	CGGTCG	7	432
CHR6	CGAGCG	CGCTCG	12	1089
CHR6	CGGCCG		16	1368
CHR7	CGAACG	CGTTCG	10	251
CHR7	CGATCG		4	98
CHR7	CGACCG	CGGTCG	10	498
CHR7	CGAGCG	CGCTCG	17	1282
CHR7	CGGCCG		25	1678
CHR8	CGAACG	CGTTCG	7	179
CHR8	CGATCG		3	67
CHR8	CGACCG	CGGTCG	7	376

CHR8	CGAGCG	CGCTCG	11	960
CHR8	CGGCCG		15	1235
CHR9	CGAACG	CGTTCG	7	233
CHR9	CGATCG		3	78
CHR9	CGACCG	CGGTCG	7	447
CHR9	CGAGCG	CGCTCG	13	1075
CHR9	CGGCCG		18	1380
CHR10	CGAACG	CGTTCG	10	176
CHR10	CGATCG		4	79
CHR10	CGACCG	CGGTCG	8	393
CHR10	CGAGCG	CGCTCG	15	1071
CHR10	CGGCCG		19	1363
CHR11	CGAACG	CGTTCG	8	268
CHR11	CGATCG		4	79
CHR11	CGACCG	CGGTCG	9	514
CHR11	CGAGCG	CGCTCG	16	1164
CHR11	CGGCCG		21	1546
CHR12	CGAACG	CGTTCG	7	189
CHR12	CGATCG		3	69
CHR12	CGACCG	CGGTCG	7	454
CHR12	CGAGCG	CGCTCG	13	894
CHR12	CGGCCG		17	1274
CHR13	CGAACG	CGTTCG	3	92
CHR13	CGATCG		1	33
CHR13	CGACCG	CGGTCG	2	214
CHR13	CGAGCG	CGCTCG	4	487
CHR13	CGGCCG		6	678
CHR14	CGAACG	CGTTCG	4	166
CHR14	CGATCG		2	45
CHR14	CGACCG	CGGTCG	4	324
CHR14	CGAGCG	CGCTCG	8	731
CHR14	CGGCCG		10	959
CHR15	CGAACG	CGTTCG	5	145
CHR15	CGATCG		2	47
CHR15	CGACCG	CGGTCG	5	339
CHR15	CGAGCG	CGCTCG	9	825
CHR15	CGGCCG		11	1082
CHR16	CGAACG	CGTTCG	13	228
CHR16	CGATCG		5	69
CHR16	CGACCG	CGGTCG	13	479
CHR16	CGAGCG	CGCTCG	26	1113
CHR16	CGGCCG		31	1583
CHR17	CGAACG	CGTTCG	15	315
CHR17	CGATCG		8	117
CHR17	CGACCG	CGGTCG	17	609
CHR17	CGAGCG	CGCTCG	33	1438
CHR17	CGGCCG		44	1932

CHR18	CGAACG	CGTTCG	3	104
CHR18	CGATCG		1	33
CHR18	CGACCG	CGGTCG	3	207
CHR18	CGAGCG	CGCTCG	5	517
CHR18	CGGCCG		7	699
CHR19	CGAACG	CGTTCG	21	377
CHR19	CGATCG		11	124
CHR19	CGACCG	CGGTCG	31	688
CHR19	CGAGCG	CGCTCG	55	1495
CHR19	CGGCCG		85	2028
CHR20	CGAACG	CGTTCG	6	148
CHR20	CGATCG		3	57
CHR20	CGACCG	CGGTCG	7	314
CHR20	CGAGCG	CGCTCG	11	703
CHR20	CGGCCG		17	980
CHR21	CGAACG	CGTTCG	2	73
CHR21	CGATCG		1	23
CHR21	CGACCG	CGGTCG	2	118
CHR21	CGAGCG	CGCTCG	3	263
CHR21	CGGCCG		5	316
CHR22	CGAACG	CGTTCG	5	134
CHR22	CGATCG		2	54
CHR22	CGACCG	CGGTCG	7	279
CHR22	CGAGCG	CGCTCG	13	618
CHR22	CGGCCG		17	915
CHRX	CGAACG	CGTTCG	5	191
CHRX	CGATCG		2	54
CHRX	CGACCG	CGGTCG	4	363
CHRX	CGAGCG	CGCTCG	8	759
CHRX	CGGCCG		11	873
CHRY	CGAACG	CGTTCG	1	20
CHRY	CGATCG		0	12
CHRY	CGACCG	CGGTCG	0	25
CHRY	CGAGCG	CGCTCG	1	66
CHRY	CGGCCG		1	98
TOTAL	CGAACG	CGTTCG	172	5019
TOTAL	CGATCG		74	1686
TOTAL	CGACCG	CGGTCG	165	10009
TOTAL	CGAGCG	CGCTCG	298	23438
TOTAL	CGGCCG		404	30770
